# Pain and mental-health scores significantly different in male and female football-players with traits of decision-reinvestment, but heart-rate-variability differences are unsupported

**DOI:** 10.1038/s41598-025-19985-6

**Published:** 2025-09-17

**Authors:** J. Pourhassan, W. Hitzl, B. Langenstein, F. Erbguth, K. Richter

**Affiliations:** 1 University Clinic for Internal Medicine, Institute for Sport Medicine, Paracelsus Medical University Nuremberg, Klinikum Nuremberg, Prof.-Ernst-Nathan-Straße 1, 90419 Nuremberg, Germany; 2 University Clinic for Psychiatry and Psychotherapy, Paracelsus Medical University Nuremberg, Nuremberg, Germany; 3 Department of Research and Innovation Management (RIM): Biostatistics and Publication of Clinical Trial Studies, Paracelsus Medical University Salzburg, Salzburg, Austria; 4 Department of Ophthalmology and Optometry, Paracelsus Medical University Salzburg, Salzburg, Austria; 5 Department of Neurology, Paracelsus Medical University Nuremberg, Nuremberg, Germany; 6 Faculty for Social Work, Technical University for Applied Sciences, Nuremberg, Germany

**Keywords:** Heart rate, Cognition, Pain perception, Mental-health, Athletes, Autonomic nervous system, Human behaviour, Pain

## Abstract

Decision-reinvestment is a tendency to consciously monitor and control performance, particularly under pressure, and associated with impaired motor-control, self-reported health concerns, and conflicting evidence with regard to cognitive performance decline. Reinvestment however has also been associated with decreased cardiac vagal activity, and alternations in heart-rate-variability (HRV) which is a reflective measure of autonomic modulations and overall athletic performance. This observational study examined the relationships among HRV, cognitive function, perceived health, and decision-reinvestment with respect to on-pitch performance. The null-hypothesis was that HRV and cognitive function do not differ between reinvestment groups, but high-reinvesters report increased health concerns. HRV of 88 football-players was recorded (5-min) using an HRV-analyser. Participants self-reported mental and physical-health (SF-36), decision-reinvestment strategy (DSRS), and were assessed for memory (Backwards Corsi), selective attention (STROOP), and cognitive flexibility (WCST). Spearman correlations and two-sample tests were used for analysis. Perceived health correlated significantly with HRV, reinvestment, and WCST perseveration errors. High-reinvesters reported more pain, diminished well-being, vitality, social functioning, and emotional capability (all *p* ≤ 0.05), but showed no group differences in HRV or cognitive function. Reinvestment is linked to poor mental-health-, and health perception is in turn associated with HRV, advocating performance-focused mental-health optimisation.

**Supplementary Information:**

The online version contains supplementary material available at 10.1038/s41598-025-19985-6.

## Introduction

Mental-health and recovery are increasingly recognised as critical components influencing athletic performance^1^, especially in high-impact sports such as association football, where both physical and cognitive demands are high. Optimal mental well-being facilitates effective decision-making, focus, and stress regulation during matches, which are essential for executing complex motor-skills under pressure. Conversely, compromised mental-health, including heightened anxiety, rumination, or poor stress coping, can impair cognitive function and increase susceptibility to performance lapses^2^. Importantly, emerging evidence links poor mental-health to a greater risk of injury in competitive athletes. A cohort study during the International Association of Athletics Federations World Championships demonstrated that athletes reporting greater pre-competition concerns or lower subjective well-being were significantly more likely to sustain injuries during competition^3^. This association underscores the role of psychological factors not only in immediate performance outcomes but also in physical-health and resilience. Recovery patterns, including quality sleep and autonomic regulation as reflected in heart-rate-variability (HRV), are fundamental to restoring cognitive and physiological resources^4^. Neglecting these aspects can exacerbate stress responses, prolong fatigue, and compromise motor-control, further increasing injury risk. Addressing these factors proactively contributes to sustaining athlete readiness and longevity in competitive environments. Therefore, comprehensive athlete management must integrate mental-health monitoring and recovery optimisation as key strategies to enhance on-pitch performance and minimise injury incidence.

Performance variability under pressure has been linked to traits of decision-reinvestment, which refers to individuals with a tendency to consciously monitor and reinvest cognitive effort in previously learned and automated motor or decision-making processes during stressful situations. Initially, identified by Masters^5^, who developed a scale to measure reinvestment in motor-skills, Kinrade et al.^6^ refined this concept by creating the Decision-Specific Reinvestment Scale (DSRS), which is a 13-item Likert scale assessing the conscious processing of past decisions and errors. Higher DSRS scores indicate greater rumination and reinvestment, traits associated with performance decline under pressure, identifying such individuals as high-reinvesters. In particular, in far-aiming sports such as football high-reinvesters show altered gaze behaviour during penalty shootouts, which contributes to a decline in motor-skills and subsequently missed penalties^7^. Further research by Laborde et al.^8^ revealed that low-reinvesters had better working memory and employ more effective coping strategies during pressured tasks. Mosley et al.^9^ provided evidence linking decision-reinvestment to HRV, showing that high-reinvesters exhibit lower vagal activity and altered autonomic responses both at rest and under pressure, yet could not support earlier associated cognitive performance differences, when examining 49 individuals at rest, during task, and after. These findings collectively suggest that decision-reinvestment influences both cognitive and physiological processes related to mental-health and recovery, although the relationship between HRV differences and recovery in high-reinvesters remains unclear.

The study of Pourhassan et al.^10^ suspected that reinvestment-related differences in HRV to correlate with deficits in regeneration status, and therefore investigated the sleep patterns of 21 male and female football athletes for 7 nights in an observational pilot study. The researchers concluded that ‘athlete reinvesters potentially benefit from exercise induced vagal activity in response to reinvestment accompanied by sympathetic activity’, as indicated by the researcher’s null hypothesis. Furthermore, the paper also claims that players with poor perceptions of their own physical-health ‘have greater tendency to ruminate and reinvest in decisions, suggesting an interrelation between reinvestment traits and physical-health’. Ironically, the researchers could not support cognitive decline in high-reinvesters either, but improved performance in terms of cognitive flexibility in individuals with poor health perceptions.

In summary, the previously discovered performance decline in reinvesters is conflicting, and the extent to which perceived health concerns and alternations in HRVs are valid risk factors, remains unknown. The primary aim of this cross-sectional study therefore was to reassess HRV and cognitive performance in an athlete population, and to seek evidence in terms of biomarkers for reinvesters poor perception of their physical-health. The hypothesises were that in an athlete population high-reinvesters (I) show no significant difference in HRV, (II) or in cognitive performance in comparison to low-reinvesters. And, that (III) perception of health is greater in low-reinvester individuals. Exploratively, the study examined whether perceived health of athletes correlates with HRV or cognitive function.

## Methods

For the purpose of this cross-sectional study which aimed to investigate HRV and cognitive performance in football athletes, with a focus on differences between high- and low-reinvesters, the methodology closely followed the protocol established by Pourhassan et al.^10^, who conducted a thorough and critical review of the chosen methods. Participant recruitment targeted a balanced number of male and female athletes from leagues one to six, using word-of-mouth and a detailed screening process to exclude confounding health factors, such as respiratory, cardiovascular, metabolic, and psychiatric conditions, as well as medication use. HRV data were collected under standardised conditions participants were seated, then rested for 10 min, and measurements were taken in a controlled environment during the competitive season with no recent long-distance travel. This approach ensures consistency and minimises external influences on physiological and cognitive outcomes, and is consistent with the CHAMP statement^11^ as well as with the Declaration of Helsinki.

### Participants

Eighty-eight football athletes of the first to sixth league were recruited via the word of mouth to participate in this research. Those were (1) males and females (2) aged 18–36 years, and (3) willing to participate in the study. Exclusion criteria were likewise the Pourhassan protocol: ‘(1) respiratory disorders, including asthma if not controlled; (2) cardiovascular issues, including uncontrolled hypertension, heart problems etc.; (3) diabetes type 1 or type 2, or any metabolic condition affecting the ability to perform sustained exercise, (4) recurring joint, bone or muscular injury or condition, (5) having had exercise testing in the previous 2 years that was terminated prematurely for reasons of health or safety, (6) any other disorder, complaint or injury disclosed to the investigators which may put the participant or an investigator at risk, or may affect the result of the study; (7) inability to participate in one or more of the tasks described in the study, or if it could be potentially detrimental to do so; (8) regular medications or used recreational drugs that might affect the results, or exacerbate a condition that is being treated; (9) cardiac pacemakers fitted, or any metallic implants (such as those used in bone surgeries etc; (10) currently undergoing or seeking treatment for a psychiatric disorder; (11) currently undergoing or seeking treatment for a medical condition that affect the ability to achieve restful sleep, such as insomnia and sleep apnoea. Participants were screened and preselected via informal interviews to ensure eligibility for inclusion. Interview was led by a general health questionnaire’^10^.

### Apparatus

An electrocardiogram (ECG) based HRV analyser Nilas MV^®^ Dinamika (CE number 1011), using Nilas Medi software, version V 5.3.2 (Nilas MV GmbH, Germany), with an HP EliteBook 820 G3. laptop, running on Intel, 2 Core i5-6200U, 2.30 GHz (turbo: 2.80 GHz), 4096 MB RAM, DDR4, 500 GB hard drive, HDD (SATA), and Windows 10 Professional 64 bit was used.

### Procedure

The participants were electronically provided a participant information sheet, which had previously been explained to them, and were instructed to ask questions at any time. After providing informed consent, the athletes had (1) rested on a chair for 10 min, and were invited to hydrate. Athletes’ wrists were then prepared with an electrode spray and connected to the HRV analyser; (2) HRV was recorded, as recommended by the manufacturer, seated, with both feet on the ground, their eyes open, watching the wall in front of them, for 300 R-R intervals. The players were subsequently asked to perform a set of questionnaires and cognitive tests on the local venue laptops via ‘the online platform PsyToolkit, which is a software package for programming psychological experiments using Linux^12,13^, and to perform the (3) Health Questionnaire SF-36^14^ to examine athletes’ general mental and physical-health status; and (4) the Decision-Specific Reinvestment Scale, to investigate decision-making strategies under pressure^6^’; as well as a (5) set of general questions with respect to exercise volume, diet, and regeneration. To control for confounding variables that can potentially influence HRV outcomes^15^, players particularly reported (a) hours after the last meal or snack (b) and last coffee or energy drink, (c) hours after the last training session, (d) smoking habits, (e) as well as years of experience in football, but also (f) how many hours of each cardio, strength and mobility training they performed during the week. ‘Questionnaires were followed by (6) the Backwards Corsi Task, to test memory function^16^; (7) the STROOP Test, to test selective attention performance, and to raise stress levels^17^; as well as the (8) the Wisconsin Card Sorting Test, to check for cognitive flexibility^18^, which because of the STOOP can be considered a pressured task.’ Evaluation took place in a 4-month total timeframe, and took place in a spear room at the venue, during the day before evening training sessions. Athletes were in-season, and had no trans-meridian travel, and were asked to arrive well rested (min. 48 h recovery after last competition), were well hydrated and had no heavy meal intake, likewise as they would pre-competitions.

### Measures

The data collected were as follows:

(A) Heart-rate-variability including an inbuilt time-frequency analysis (Table 1).
(B) Perceived health.
  1. Each for SF-36 physical-health scores,
  2. As well as the SF-36 mental-health scores;

**Table 1:** Parameters of the Nilas MV^®^ Dinamika HRV analyser.

Parameters of HRV	Definition
(I) Parameters heart activity	(1) Heart rate (HR): Heart rate measured in beats per minute (bpm)
	(2) Inspiratory amplitude band (IAB): Represents heart rate variations during the inspiratory phase of breathing
	(3) Very inspiratory heart rate (VIHR): Represents heart rate variations specifically during the inspiratory phase of breathing
	(4) Inspiratory amplitude range (IAR): Measures the range of heart rate variations during the inspiratory phase
	(5) Baevsk’s stress index (SI): Index indicating stress levels based on HRV
(II) Parameters vegetative regulation	(6) B1 (%): Percentage of power in the very low-frequency range (VLF) indicating various regulatory mechanisms
	(7) B2 (%): Percentage of power in the low-frequency range (LF) associated with sympathetic and parasympathetic activity
(III) Statistical parameters	(8) R-R intervals (RRNN): Duration between successive R peaks in milliseconds (ms)
	(9) Standard deviation of R-R intervals (SDNN): Measures the overall variability of the heart rate in ms
	(10) CV (Coefficient of Variation): Represents the ratio of the standard deviation to the mean of RR intervals, indicating the relative dispersion of heartbeats (%)
	(11) Root mean square of successive differences (RMSSD): Reflects short-term HRV in ms
	(12) Mean number of times in which the change of R-R intervals exceeds 50 ms (NN50)
	(13) Mean percentage of times the change of R-R intervals exceeds 50 ms (pNN50): %
(IV) Frequency spectrum	(14) High-frequency power (HF): Power in the frequency range of 0.15 to 0.4 Hz (ms²)
	(15) Low-frequency power (LF): Power in the frequency range of 0.04 to 0.15 Hz (ms²)
	(16) Very low-frequency power (VLF): Power in the frequency range of 0.0033 to 0.04 Hz (ms²)
	(17) HFnu: Normalised high-frequency power
	(18) LFnu: Normalised low-frequency power
	(19) LF/HF Ratio: The ratio of LF power to HF power
	20) Total frequency power (TP): Total power in the frequency domain (ms² * 1000)
(V) Parameters histogram	21) Mode (Mo): Most frequent R-R interval in ms
	22) Percentage of Mode Amplitude (AMo): %
	23) Ventricular response (VR): Represents the heart’s response to different stimuli, used in arrhythmia detection
	24) HRV index: A composite index summarizing various HRV parameters and indicating overall heart-rate-variability (ms)
(VI) Scatterogram	25) Standard deviation perpendicular to the line of identity (SD1): ms
	26) Standard deviation along the line of identity (SD2): ms
	27) Ratio of SD2 to SD1 (SD2/SD1)
(VII) Parameters autocorrelation	28) 1-k second correlation (1k): Measures the correlation between RR intervals at a given time and those occurring k seconds earlier
	29) Mean of zero crossings (m0Z): Represents the mean number of zero crossings in the RR interval signal
	30) Interquartile correlation (IC): Measures the correlation between RR intervals within the interquartile range

Physical Functioning (SF-36 questions 3–12), was not assessed, as athletes had been in-season and were otherwise excluded (Table 2).

(C) Decision-reinvestment (DSRS).
  1. Rumination;
  2. Reinvestment;
  3. Overall score;
(D) Cognitive function.
  1. Backwards Corsi: best score;
  2. STROOP: number of successful attempts;
  3. Wisconsin Card Sorting Test (WCST): number of perseverance errors.

**Table 2:** As per 'the usage of a software to process the SF-36 data’ all items except #2 which is considered Transitional^34^.

Items	Scale	Summary measure
3. Vigorous activities	Physical functioning	Physical-health
4. Moderate activities		
5. Lift, carry groceries		
6. Climb several flight		
7. Climb one flight		
8. Bend, kneel		
9. Walk mile		
10. Walk several blocks		
11. Walk one block		
12. Bathe, dress		
13. Cut down time	Capability physical	
14. Accomplish less		
15. Limited in kind		
16. Had difficulty		
21. Pain magnitude	Bodily pain	
22. Pain interfere		
1. Rating	General health	
33. Sick easier		
34. As healthy		
35. Health to get worse		
36. Excellent health		
23. Pep/life	Vitality	Mental-health
27. Energy		
29. Worn out		
31. Tired		
20. Social extend	Social functioning	
32. Social time		
17. Cut down time	Capability emotional	
18. Accomplish less		
19. Not careful		
24. Nervous	Well-being	
25. Down in dumps		
26. Peaceful		
28. Blue/sad		
30. Happy		

### Data analysis

The data were checked for consistency and normality. Fisher’s Exact test or Pearson’s Chi-Square test was used to analyse cross-tabulations. Randomisation tests with and without assumption of variance homogeneity were performed to evaluate means between two independent groups, as well as modified Levene’s test to specify variances between groups. Furthermore, Kruskal–Wallis ANOVA was applied to compare medians. To test the effect of age on binary variables generalised linear models based on the binomial distribution and the logit as a link function were carried out. Finally, Spearman correlations with corresponding tests were used to analyse correlations between continuous variables. All reported tests were two-sided, and the significance levels were set at *r* ≥ 0.5, and *p* ≤ 0.05. All the statistical analyses in this study were performed via STATISTICA 13 (Hill, T. & Lewicki, P. Statistics: Methods and Applications. StatSoft, Tulsa, OK) and NCSS (NCSS 2022, NCSS, LLC. Kaysville, UT). Participants with total DSRS scores of ≤ 26 were assigned to the low-reinvesters group, and participants with scores ≥ 27 were assigned to the high-reinvesters. Moreover, this method was also applied to both the rumination (7 statements) and reinvestment (6 statements) aspect. Therefore, low-rumination was cut at ≤ 14, and low-reinvestment at ≤ 12^10^. Due to the explorative nature of the study, no prior sample size computation was carried out, and an aposteriori sample computation with the primary endpoint RMSSD^8^ was performed; as well as an equivalence test of means using two one-sided equal-variance t-tests with sample sizes of low-reinvester group vs. high-reinvester group.

### Ethics

Ethical concerns only existed for the STROOP test in regard to minor psychological manipulations, which were addressed with a preselection and exclusion of participants who could be possibly affected by underlying conditions, by performing a general health questionnaire upon recruitment. The project was conducted under the academic ethical framework of the Paracelsus Medical University and Klinikum Nuremberg, and was registered under the reference number FMS_W_140.22-PMU to obtain a waiver.

### EDI statement

Furthermore, the interdisciplinary research team aimed to assess a balanced number of male and female athletes for this study, and consisted of physiology, cardiology, sport medicine, neurology, psychiatry and data science experts.

### Patient and public involvement

Finally, there was neither patient nor public involvement.

## Results

The data of all male (*N* = 48) and female (*N* = 40) participants were taken into account. Athletes were 24 years old (SD 4), with a seated heart-rate of 67 bpm (SD 11); and a Total Frequency Power of 4.991 Hz (SD 4221). Individuals arrived with 32 h (SD 18) of regeneration on average, although *N* = 4 players only had a recovery time of ≦ 6 h. Extra hydration before testing was rejected by 31 participants, and each 1 player in the first, third and sixth league (*N* = 3) was a smoker. There were 50 (26 males) high- and 38 (22 males) low-reinvesters, but they were not normally distributed. The equivalence test of means via two one-sided equal-variance t-tests with sample sizes of 38 in the low-reinvester group and 50 in the high-reinvester group achieves 80% power at the 5% significance level. Whereby, equivalence was defined when difference between both means was < 19.5, at the − 19.5 and 19.5 equivalence limits. The test confirms the equivalence (*p* = 0.005) of RMSSD in both the high- and low-reinvester groups, the actual difference between the means is 2.92, and the standard deviation is 29.5 in each group.

**Table 3:** Correlation matrix of the correlation coefficient (r) and probability (p).

Correlation matrix	Physical-health		Mental health							
	SF-36 general health		SF-36 emotional capability		SF-36 well-being		SF-36 social function		SF-36 vitality	
**Personality traits**	*r*	*p*	*r*	*p*	*r*	*p*	*r*	*p*	*r*	*p*
Decision-reinvestment			− 0.25	0.021	− 0.48	0.000	− 0.36	0.001	− 0.36	0.001
**HRV**										
Heart rate (HR): Heart rate measured in beats per minute (bpm)	− 0.33	0.002	− 0.23	0.031						
Inspiratory amplitude band (IAB): Represents heart rate variations during the inspiratory phase of breathing	− 0.34	0.001	− 0.24	0.023						
Inspiratory amplitude range (IAR): Measures the range of heart rate variations during the inspiratory phase	− 0.40	0.000	− 0.29	0.006						
Baevsk’s stress index (SI): Index indicating stress levels based on HRV	− 0.38	0.000	− 0.27	0.010						
B1 (%): Percentage of power in the very low-frequency range (VLF) indicating various regulatory mechanisms	0.37	0.000	0.25	0.021						
B2 (%): Percentage of power in the low-frequency range (LF) associated with sympathetic and parasympathetic activity	0.21	0.049								
R–R intervals (RRNN): Duration between successive R peaks in milliseconds (ms)	0.33	0.002	0.23	0.029						
Standard deviation of R–R intervals (SDNN): Measures the overall variability of the heart rate in ms	0.33	0.002	0.24	0.026						
CV (coefficient of variation): Represents the ratio of the standard deviation to the mean of RR intervals, (%)*	0.21	0.050								
Root mean square of successive differences (RMSSD): Reflects short-term HRV in ms	0.33	0.002	0.28	0.008						
Mean number of times in which the change of R–R intervals Exceeds 50 ms (NN50)	0.30	0.004	0.26	0.015						
Mean percentage of times the change of R–R intervals exceeds 50 ms (pNN50): %	0.32	0.002	0.27	0.010						
High-frequency power (HF): Power in the frequency range of 0.15 to 0.4 Hz (ms²)	0.30	0.004	0.27	0.012						
Low-frequency power (LF): Power in the frequency range of 0.04 to 0.15 Hz (ms²)	0.23	0.029								
Very low-frequency power (VLF): Power in the frequency range of 0.0033 to 0.04 Hz (ms²)	0.29	0.006			0.25	0.019	0.23	0.034		
Total frequency power (TP): Total power in the frequency domain (ms^2^ × 1000)	0.33	0.002	0.25	0.020						
Mode (Mo): Most frequent R–R interval in ms	0.32	0.003	0.25	0.017						
Percentage of mode amplitude (AMo): %	− 0.34	0.001	− 0.24	0.022						
Ventricular response (VR): Represents the heart’s response to different stimuli, used in arrhythmia detection	0.32	0.002	0.22	0.038						
HRV index: A composite index summarizing various HRV parameters and indicating overall heart-rate-variability (ms)	0.34	0.001	0.25	0.020						
Standard deviation Perpendicular to the Line of Identity (SD1): ms	0.33	0.002	0.28	0.008						
Standard deviation Along the Line of Identity (SD2): ms	0.33	0.002	0.24	0.027						
Mean of zero crossings (m0Z): Represents the mean number of zero crossings in the RR interval signal	0.22	0.041								
**Cognitive function**										
Perseveration errors WCST									0.27	0.013
**Exercise**										
Strength training/week (h)									0.24	0.025
Endurance training/week (h)			0.32	0.002						

*Indicating the relative dispersion of heartbeats.

Decision-reinvestment is negatively correlated with all 4 parameters of mental-health, namely: emotional capability, well-being, social functioning, as well as vitality (Table 3). Parameters of heart activity, decrease with increasing perceived health and emotional capability scores, whereas vegetative regulation increases. Additionally, HRV statistics, as well as frequency powers, measures of Histogram, Scatterogram, and Autocorrelation significantly correspond with perception of both physical- and mental-health, as likewise displayed in Table 3. A positive correlation further existed with respect to mental-health with hours of strength training and was even more significant with hours of endurance training; as well as in both vitality and preservation errors in the WCST (Table 3).

Further analysis revealed that pain scores were significantly greater in high-reinvesters (p 0.042) in comparison to low-reinvesters. Reinvestment-related group differences also occur with respect to vitality (p 0.001), social functioning (p 0.001), emotional capability (p 0.046) as well as well-being (*p* < 0.001). Moreover, no evidence was found for reinvestment-based group differences related to cognitive function or HRV either. The HRV norm values are provided in the Appendix in Table 4.

Additional analyses revealed, gender was not considered a confounding factor in this study. In the context of this study, gender did not moderate reinvestment-related group differences in HRV, health indicators, or cognitive outcomes. Although it is well-established that baseline HRV values typically differ between males and females, with females generally showing higher resting heart rates and distinct autonomic patterns, these physiological differences did not affect the present findings. Importantly, both high- and low-reinvester groups were balanced in terms of gender, ensuring that any inherent sex-related HRV variations were evenly distributed and therefore not relevant to the observed reinvestment-related outcomes.

In summary, decision-reinvestment traits were linked to poorer perceived mental and physical-health, despite no measurable group differences in HRV or cognitive performance. These findings suggest that psychological variables, may critically shape health perceptions in high-performance athletes.

## Discussion

This study aimed to explore the relationships among decision-reinvestment, perceived health, cognitive performance, and HRV in an athletic population. Given the heightened cognitive and physiological demands placed on competitive athletes, understanding how individual psychological traits such as reinvestment affect health and performance outcomes is of both scientific and practical relevance. This research focused on determining whether high-reinvesters differ from low-reinvesters in HRV, cognitive performance, and health perception. The hypothesis that in an athlete population high-reinvesters (I) show no significant difference in HRV, (II) or in cognitive performance in comparison to low-reinvesters, and that (III) the perception of health is greater in low-reinvesters, but does not correlate with any HRV or cognitive function, can be confirmed. First, reinvestment is correlated with mental-health only, but not with any parameters of physical-health such as general health, physical capability or pain scores; however further analysis revealed that high-reinvesters had significantly greater pain scores than low-reinvesters did. Second, the perception of general health corresponds to all seven HRV parameters, whereas emotional capability is linked to all but the parameters of autocorrelation. Finally, physical-health concerns did not correlate with cognitive function, but interestingly individuals with superior mental-health perceptions, particularly with respect to vitality, had an increase in preservation errors during the WCST, partly supporting the theory that individuals with poor health perceptions have increased cognitive flexibility^10^. Although, the researchers have previously claimed that physical-health is correlated with athletes’ error-rates during the WCST, the current data suggest that mental-health, particularly mental fatigue (vitality), is the key driver of cognitive flexibility in athletes.

However, a minor limitation of the study was that typical background noise on the venue outside the testing area, that could have potentially impacted attention as well as breathing patterns and consequently altered the HRV^15^. Nevertheless, testing took place in participants’ natural habitat and therefore might overcome the limitations of a clinical setting in regard to implication of the outcomes with respect to pitch performance^19^. Another restraint was the testing computers at the pitch. Owing to the variety of laptops provided for testing cognitive function, reaction-times were excluded from the data set, especially as some participants discovered the significantly faster touch-screen functions on some devices, whereas others continued using the mousepad. Additionally, regeneration status in terms of hours of recovery from the last training session to testing varied severely. While recovery-related variables such as hydration, and recent physical activity were considered during study design, variability in individual recovery states may have introduced noise into HRV results. However, variables such as bodyweight, alcohol, sleep as well as individual’s estimated personal regeneration demands were not assessed in this study. This limitation should be addressed in future studies with tighter control over recovery status. HRV is significantly influenced by circadian rhythm, body weight, physical activity, and other lifestyle factors^15^. According to the manufacturer the accuracy of the Nilas MV^®^ system was validated against a gold-standard ECG simulator (SIM-01, Labtech, Hungary) using a reference output of 60 beats per minute. The resulting R–R intervals across all tested Nilas MV devices consistently matched the expected 1000 ms interval, indicating high consistency and reliability under controlled conditions. In October 2017, parallel measurements with a chest-strap system (FitBit) and Nilas MV confirmed concordance of heart rate data in resting conditions. To date, no formal validation study has been conducted comparing the Nilas MV^®^ system to the clinical gold standard of ECG-derived HRV with high-resolution R–R interval detection and spectral analysis using Fourier or autoregressive methods, limiting direct comparability with established benchmarks.

### Perceived health, cognitive flexibility and reinvestment

Even though reinvestment per se did not correlate with any aspects of cognitive function at all in this research^9^, a paradoxon that could be reproduced in group comparisons is that athletes with lower health perceptions, particularly lower vitality, have increased cognitive flexibility^10^. Unlike prior research mental-rather than physical-health seem to determine outcomes in the WCST. Compared with individuals who reported lower mental-health, individuals who reported better mental-health had decreased cognitive flexibility as indicated by significantly more preservation errors. However, vitality as well as all other measures of mental-health correlated with decision-reinvestment and are also significantly lower in high-reinvester individuals than in low-reinvestment scorers. In this respect, the study of Hotopf et al.^14^ evaluated the usefulness of the SF-36 for detecting depressive and anxiety disorders, and concluded that both lower well-being and overall mental-health scores increase the likelihood of the presence of probable major depressive disorders. Although high-reinvester athletes are far from having low total scores, low-reinvesters tend to have significantly higher mental-health scores. While the correlation of decision-reinvestment solely relies on estimated mental- rather than physical-health, pain estimations on the other hand are significantly greater in high-reinvesters in group comparison against low scorers. However, a key attribute of reinvesters, is that individuals do not stick to acquired and automated behaviour under pressure^6,7,9^. Henceforth, it is suspected that reinvestment behaviour could be triggered by stress-induced mental fatigue, rather than by traits alone. The STROOP test, which was performed prior to the WCST in this study, is a validated stressor that increases cortisol secretion^20^, retards the dorsolateral prefrontal cortex^21^, and consequently increases mental fatigue. Individuals who rate their vitality greater (lower mental fatigue) do not alter their response despite errors and increased cortisol levels, as indicated by the number of preservation errors in the WCST. Whether mental fatigue potentially induces reinvestment behaviour remains unknown at this point and certainly demands further empirical validation. Athletes with greater mental fatigue, as well as high-reinvesters, might benefit the most from mental-health support, with respect to pressured task execution on the pitch^22^. The fact that vitally is significantly lower in high-reinvesters, is not reflected in the cognitive performance outcome of high-reinvesters in the selected test battery.

### HRV, pain, and mental-health

Nevertheless, pain scores do not interact with any HRV parameters whatsoever. While acute pain can manifest as alterations in HRV, the complex relationship between pain and HRV still relies on the intensity of pain, individual differences in pain perception, and psychological factors, and therefore may not always be the sole determinant of HRV changes^23^. It should also be noted that this study did not examine whether elevated pain reports reflect only true physiological sensitivity or cognitive-emotional biases such as catastrophising or attentional bias. Distinguishing between these mechanisms in further research would provide greater clarity regarding whether reinvestment is primarily associated with heightened nociception or with maladaptive appraisal of pain. Mediating factors as such have not been assessed in this paper, and future studies could benefit from incorporating pain threshold assessments alongside self-report measures. On the other hand, pain is considered a stressor and, as a result, triggers the same neurological fight or flight path as any other aggravation^24^. Interestingly, the research of Williamson concluded that the anticipatory response to stress and pain uses the same neuroanatomical structure as exercise effort does. A crucial part of a healthy stress response is that, after the stressor is removed, there is a recovery phase^25^. Stress that is not addressed impacts metabolism, digestion, as well as wound healing, and can even lead to tissue damages on the long run^25^. Unlike stress, which requires physical activity to be fought or fled for a healthy response, exercise inevitably demands physical activity, and may even enhance physiological processes, such as HRV^26^, if performed within the scope of supercompensation^27^. Therefore, prescribed exercise might reduce not only stress^26^, but also pain perception^28^, through the use of the same neuroanatomical structures of a fight-flight response^24^. However, high-reinvester athletes tend to perceive their pain greater pain in comparison to low-scorers, even though HRV measures do not correspond. Consequently, reduced mental-health scores in high-reinvesters could be a result of individuals’ perceived pain. It is therefore possible that aerobic capacity or training-induced autonomic resilience buffers HRV responses in well-trained athletes. Whether the pain perceptions of non-athlete reinvesters are the same and whether HRV parameters differ from those of athletes with respect to pain remain unknown. Additionally, it remains unclear whether reinvestment drives these effects or if underlying psychological factors, such as anxiety or stress, contribute to both reinvestment tendencies and poorer health outcomes. Accordingly, longitudinal or experimental designs would be necessary to disentangle these relationships in further research. A hypothesis could be that minor perceptions of pain may not result in altered HRV to the same extent, as aerobic capacity may help stabilise pain-related HRV fluctuations in better trained reinvesters. Furthermore, no evidence was found for reinvestment-based group differences related to HRV. Despite prior research that has suggested reinvestment related HRV alternations^9^ in an athlete population, differences do not occur in this research, even though high reinvestment scorers perceive greater pain and show reduced mental-health. However, the Mosley study used chest electrode-based technology to assess HRV, whereas this research worked with wrist electrodes, and methodological differences as such might therefore account for discrepancies.

Moreover, athletes with greater health perception, both emotional capability and general health, tend to show enhanced vagal activity, HRV statistics, as well as frequency powers, measures of Histogram, Scatterogram, and Autocorrelation. Parameters of heart activity such as SI, inspiration and resting HR decline with increasing health perception, and an increasing Mo combined with a decreasing AMo pattern suggests a broadening of the distribution of R–R intervals, with less concentration around the most frequent interval and more variability^29^. Overall, HRV parameters improve with the perception of individuals’ health.

While HRV has emerged as a robust marker of autonomic regulation and prefrontal inhibitory capacity, it is also highly context-sensitive. Several lifestyle and physiological factors including physical fitness, hydration status, sleep quality, and recovery state have been shown to meaningfully shape HRV and vagal tone, and thus may partly explain inconsistencies across studies. For example, higher levels of cardiorespiratory fitness are consistently associated with increased vagal activity and greater resting-state HRV, reflecting a more flexible and adaptive autonomic system. Conversely, dehydration reduces plasma volume and increases cardiovascular strain, which can suppress parasympathetic activity and reduce HRV. Similarly, sleep quality and quantity play a crucial role in autonomic balance: poor or restricted sleep is linked to reduced vagal modulation, impairing both safety learning and fear extinction processes. Recovery status also exerts strong effects, as accumulated fatigue or insufficient recovery after stress or exertion can blunt vagal tone and lower HRV^30^.

These findings suggest that individual differences in HRV may not only reflect trait-like predispositions (e.g., anxiety vulnerability, inhibitory control) but also dynamic state-dependent influences that fluctuate with daily health and lifestyle conditions. Consequently, variability in physical fitness, hydration, sleep, and recovery across participants and across studies may contribute to divergent results in the fear-conditioning literature. Recognising and, where possible, controlling for these contextual influences is essential for accurate interpretation of HRV data, particularly in paradigms that rely on vagal modulation as a marker of fear learning and extinction.

### Vitality, emotional capacity and exercise

Finally, a reduction in mental fatigue and increase in vitality seem to correspond with hours of strength training, whereas emotional capability increases with hours of endurance training, supporting the theory that exercise increases mental-health^26^, even in an athlete population.

### Clinical implications

Reinvester athletes require particular clinical attention to address anxiety-causing symptoms, which might threaten both task execution^6^ and injury risk^3^ during competitions. The data adds to those of Birrer et al.^31^ who suggested that reinvestment derives from a lack of emotion control training, which was later confirmed by Sparks^32^ who introduced mindful awareness training as a ‘moderator of reinvestment on the anxiety-performance relationship’. Recent studies have shown that awareness training, such as Mindfulness-Based Stress Reduction (MBSR), may also improve chronic pain perception^33^ and may be even more beneficial in reinvester athletes, as individuals have both reduced mental-health scores and greater pain scores. The pain perception of high-reinvesters, however may not be linked to HRV measures and requires further investigation. Further research is warranted to clarify whether reinvestment is a stable trait or modifiable through mental fatigue and stress. Experimental studies could assess HRV and cognitive responses in reinvesters vs. non-reinvesters under controlled stress conditions. Additionally, comparisons between athletic and non-athletic populations could help isolate the influence of training-induced autonomic regulation on pain perception and HRV variability. Longitudinal studies may also reveal how changes in training volume or mental-health influence reinvestment behaviour over time.

## Conclusion

This study provides new insights into the complex relationships among reinvestment, perceived health, cognitive performance, and HRV in athletes. Overall, the data did not provide evidence for reinvestment-related differences in baseline HRV. Additionally, a relationship between reinvestment and cognitive performance cannot be supported in endurance athletes. However, individuals with higher reinvestment scores reported greater pain perceptions and lower mental-health scores. Pain perceptions, however, showed no association with any HRV parameters. Interestingly, mental-health correlated with cognitive flexibility in the WCST, suggesting that reinvestment tendencies may be exacerbated by mental fatigue, independent of trait levels. Furthermore, the number of hours dedicated to both strength and endurance training was significantly associated with mental-health, even among football-players, while poorer mental-health was linked to higher decision-specific reinvestment. Notably, HRV measures in this study corresponded only with athletes’ physical and emotional health, without any apparent interaction with cognitive performance. These findings suggest that clinical and training interventions aimed at enhancing mental resilience and recovery could be effective in managing reinvestment tendencies in athletes.

## Supplementary Information

Below is the link to the electronic supplementary material.

Supplementary Material 1

## Data Availability

The original contributions presented in the study are included in the article Supplementary material, further inquiries can be directed to the corresponding authors.

## Appendix

**Table 4:** HRV norm values according to NILAS MV^®^ HRV analyser.

Parameters of HRV		Norm
(I) Parameters heart activity	HR (beats/min)	[60–90]
	IAB	[35.0-145.0]
	VIHR	[0.25–0.60]
	IAR	[15.0–50.0]
	SI	[10.0-100.0]
(II) Parameters vegetative regulation	В1 (%)	[60–100]
	В2 (%)	[60–100]
(III) Statistical parameters	RRNN (ms)	[700–1000]
	SDNN (ms)	[30.0-100.0]
	CV (%)	[3.0–12.0]
	RMSSD (ms)	[15.0–45.0]
	NN50	[46–100]
	pNN50 (%)	[15–34]
(IV) Frequency spectrum	HF (ms²)	[770–1078]
	LF (ms²)	[754–1586]
	VLF (ms²)	[600–1500]
	HFnu	[26.00–32.00]
	LFnu	[50.00–58.00]
	LF/HF	[1.50-2.00]
	TP (ms²*1000)	[2385–4545]
(V) Parameters histogram	Mo (ms)	[700–900]
	AMo (%)	[30.00–50.00]
	VR (ms)	[150–450]
	HRV Index	[20.0–50.0]
(VI) scatterogram	SD1	[10.0–40.0]
	SD2	[50.0-121.0]
	SD2/SD1	[1.2-4.0]
(VII) Parameters autocorrelation	1k	[0.700-1.000]
	m0	[0–18]
	Z	[50.0-500.0]
	IC	[1.0–3.0]

## Declarations

### Prior presentation

This manuscript is based on work that has previously been presented at the 10th Congress of the European Academy of Neurology in Helsinki, Finland (2024), with its abstract published under the reference EPR-047 in the conference issue 10.1111/ene.16338.
